# Should all pregnant women take calcium supplements in Nepal? GRADE evidence to policy assessment

**DOI:** 10.1080/16549716.2022.2128283

**Published:** 2022-10-14

**Authors:** Khem Narayan Pokhrel, Saki Thapa, Paul Garner, Maxine Caws, Raghu Dhital, Suman Chandra Gurung, Tilly Fox, Samjhana Shrestha

**Affiliations:** a READ-It Project, Birat Nepal Medical Trust, Kathmandu, Nepal; b Centre for Evidence Synthesis in Global Health, Liverpool School of Tropical Medicine, Liverpool, UK; c Department of Clinical Sciences, Centre for Drugs and Diagnostics, Liverpool School of Tropical Medicine Liverpool

**Keywords:** Calcium supplementation, pregnant women, pre-eclampsia, GRADE, Nepal

## Abstract

**Background:**

The WHO recommends oral calcium supplementation (1.5–2.0 g) in pregnant women to reduce the risk of pre-eclampsia living in areas with low dietary calcium intake. Although maternal mortality is high in Nepal and eclampsia causes at least 20% of maternal deaths, implementing WHO recommendations would be a major undertaking.

**Objective:**

This review aimed to assess whether the current evidence supports the blanket supplementation of calcium to prevent pre-eclampsia among pregnant women in Nepal.

**Methods:**

We used a structured approach to appraise the evidence for calcium supplementation in Nepal. We identified what may influence the impact of calcium supplementation in Nepal and conducted a situation analysis in the country covering maternal mortality, pre-eclampsia occurrence, and existing government policy provisions for supplementation. We also consulted with experts and government officials to explore their perspectives and experience on supplementation. We then used AMSTAR (A MeaSurement Tool to Assess Systematic Reviews) to appraise the Cochrane Systematic Review of calcium supplementation. Finally, we used these data in a GRADE (Grading of Recommendations Assessment, Development and Evaluation)–Evidence to Decision framework to reach a policy recommendation.

**Results:**

Our assessment of the Cochrane Review showed that the recommendation made by the WHO is based on weak evidence and trial findings that are not consistent between studies. The Cochrane Review found low certainty of the evidence for benefit (reduction in pre-eclampsia and maternal mortality). Conversely, there is a high certainty of the evidence of undesirable effects (HELLP [haemolysis, elevated liver enzymes and low platelets] syndrome) although this is uncommon. The likely absolute reduction in maternal deaths projected to Nepal was estimated to be low, while the implementation costs were high. Stakeholders also raised several concerns regarding feasibility, acceptability, appropriate dosing, and risk communication.

**Conclusions:**

This review concludes that the blanket supplementation of calcium cannot be recommended in Nepal. A better approach may be to identify high-risk pregnant women and manage their antenatal visits and delivery to prevent mortality from pre-eclampsia.

## Background

### Introduction

The World Health Organization (WHO) currently recommends oral calcium supplementation (1.5–2.0 g) for pregnant women in areas with low dietary calcium intake to reduce the risk of pre-eclampsia [1]. This recommendation is based on the 2018 Cochrane systematic review which concluded that there is a 55% reduction in the overall risk of pre-eclampsia in calcium-supplemented pregnant women and a 64% risk reduction in pregnant women with a low-calcium diet [2]. However, these estimates were of low certainty due to variation in effect sizes between different trials.

Reducing maternal mortality remains a priority in Nepal, where 239 maternal deaths per 100,000 live births were reported in 2016 [3]. The Sustainable Development Goal target is less than 70 per 100,000 by 2030 [4]. An important cause of maternal death in Nepal is eclampsia, which can be prevented through the effective detection and management of pre-eclampsia. The extensive 2009 Maternal Mortality and Morbidity Study for Nepal showed that eclampsia was responsible for 21% of all maternal deaths and 29% of hospital maternal deaths in 2008/2009 [5]. The survey also reported that 12.3% of pregnant women attending hospitals were diagnosed with pre-eclampsia and 17.5% were diagnosed with eclampsia. Meanwhile, the prevalence was comparatively lower (pre-eclampsia: 7 per 1,000 live births and eclampsia: 5.5 per 1,000 live births) at peripheral health facilities [5]. Despite this, there is currently no routine screening for pre-eclampsia with blood pressure estimates or urine tests during antenatal care (ANC).

The Government of Nepal conducted a pilot evaluation of calcium supplementation for pre-eclampsia (1 g of calcium daily) in Dailekh district in 2016 [6]. This pilot study assessed the feasibility and acceptability of calcium supplementation but did not examine the effect of supplementation on the incidence of pre-eclampsia among pregnant women or mortality due to a limited sample size.

Following the WHO recommendation, calcium supplementation has been recommended in the major health policy documents of Nepal, including the National Medical Standard for Maternal and Newborn Care 2020 [7], Volume III, National Nutrition Strategy 2020, and Nepal Safe Motherhood and Newborn Health Road Map 2030 [8]. Despite this, there are no clear guidelines or implementation plans for calcium supplementation in the country. Given the significant resource requirements for such a programme, our study aimed to apply GRADE (Grading of Recommendations Assessment, Development and Evaluation) and the Evidence to Decision (EtD) framework to review the global evidence base regarding blanket calcium supplementation in pregnant women in the context of Nepal and make an updated, locally appropriate policy recommendation.

#### Research question

Does the current evidence support blanket supplementation of calcium to prevent pre-eclampsia among pregnant women in Nepal?

## Methods

We applied five steps to appraise the evidence regarding calcium supplementation in pregnant women in Nepal. First, we identified relevant factors known or likely to influence the impact of calcium supplementation in the Nepali context, such as dietary calcium intake levels, the prevalence of pre-eclampsia among women, and existing supplementation programmes targeted to pregnant women such as iron/folic acid tablets. We then conducted a situation analysis reviewing the available data on maternal mortality, pre-eclampsia, and related programmes and plans targeted towards calcium supplementation in Nepal. In the third stage, we conducted a critical appraisal using the AMSTAR (A MeaSurement Tool to Assess Systematic Reviews) checklist [9] of the 2018 Cochrane systematic review [2] which was used as an evidence base by the WHO for recommending calcium supplementation for pre-eclampsia prevention in pregnant women.

We then conducted a stakeholder consultation on calcium supplementation with five specifically selected national experts and policymakers (section chief and two senior public health officers from Safe Motherhood Section, Family Welfare Division of Ministry of Health and Population (MoHP), section chief at Nutrition Section, Family Welfare Division, MoHP, and a past president of Nepal Society of Obstetricians and Gynecologists). We considered different domains of the EtD framework [10] such as desirable effects of supplementation; undesirable effects or harms; the certainty of the evidence according to GRADE; values relevant to Nepal and the specific context in which supplementation takes place such as feasibility; and resource implications. We also conducted key informant interviews by telephone with two provincial public health officials of Sudurpaschim Province, who were directly involved in the implementation of calcium supplementation programme under the family welfare division of MoHP. Phone interviews were recorded and transcribed, and a narrative synthesis prepared and interpreted, primarily focusing on their implementation experience and possible reason for discontinuation of the supplementation.

We finally evaluated the collated evidence, applying the GRADE–EtD framework to formulate a policy recommendation [11]. We present the findings below following the EtD framework format.

## Results

### Situational analysis

#### Current WHO guidance

The current WHO guideline recommends calcium supplementation (high dose 1.5–2.0 g) with oral elemental calcium for pregnant women in areas with low dietary calcium intake to reduce the risk of pre-eclampsia [1]. The research evidence for this recommendation was derived from the Cochrane systematic review (13 studies, 15,730 women; risk ratio (RR) 0.45, 95% confidence interval (CI) 0.31–0.65) [2] which showed a 55% reduction in the average risk of pre-eclampsia in pregnant women receiving calcium supplements. Subgroup analysis, however, showed a risk reduction in women with a low-calcium diet (64%) and those with a high risk of pre-eclampsia (78%), but not those with adequate calcium diets.

## Formal assessment by GRADE–EtD framework

Table 1 shows a summary of the judgements reached, considering the evidence under each criterion of the EtD framework.

**Table 1.** Summary of judgements reached under each category of EtD framework.

Title description	Judgement						
**Problem**	No	Probably no	Probably yes	Yes		Varies	Don’t know
**Desirable effects**	Trivial	**Small**	Moderate	Large		Varies	Don’t know
**Undesirable effects**	**Large**	Moderate	Small	Trivial		Varies	Don’t know
**Certainty of evidence**	Very low	**Low**	Moderate	High			No included studies
**Values**	Important uncertainty or variability	Possibly important uncertainty or variability	Probably no important uncertainty or variability	**No important uncertainty or variability**			
**Balance of effects**	Favours the comparison	**Probably favours the comparison**	Does not favour either the intervention or the comparison	Probably favours the intervention	Favours the intervention	Varies	Don’t know
**Resources required**	**Large costs**	Moderate costs	Negligible costs and savings	Moderate savings	Large savings	Varies	Don’t know
**Certainty of evidence of required resources**	**Very low**	Low	Moderate	High			No included studies
**Cost-effectiveness**	Favours the comparison	Probably Favours the comparison	Does not favour either the intervention or the comparison	Probably favours the intervention	Favours the intervention	Varies	**No included studies**
**Equity**	Reduced	**Probably reduced**	Probably no impact	Probably increased	Increased	Varies	Don’t know
**Acceptability**	No	Probably no	Probably yes	**Yes**		Varies	Don’t know
**Feasibility**	No	**Probably no**	Probably yes	Yes		Varies	Don’t know

Judgement for each category is indicated in bold text.

### Problem (is the problem a priority?)

The principal aim of calcium supplementation in pregnant women is to reduce the incidence of pre-eclampsia and therefore maternal mortality. To quantify the likely absolute effect for Nepal, we applied the effect estimate from the 2018 Cochrane Review [2] to data from the 2009 Nepal Maternal Mortality Study [5]. Reducing maternal deaths is a priority for Nepal under Sustainable Development Goal 3 [4], reflected in multiple government policy documents [7,8].

Currently, there is no routine pre-eclampsia screening incorporated into ANC in health facilities in Nepal[5]. As a result, reducing the incidence of pre-eclampsia and identifying effective interventions to achieve the maternal mortality target of less than 70 per 100,000 live births can be considered a priority for Nepal [4]. Therefore, the judgement is YES, the problem is a high priority for Nepal.

### Desirable effects (how substantial are the desirable anticipated effects?)

The desirable effects are a reduction in pre-eclampsia and maternal mortality.

Although the available evidence suggests that high-dose calcium supplementation may reduce pre-eclampsia in pregnant women (RR: 0.45, 95% CI 0.31–0.65) and women with low-calcium diet (RR: 0.36, 95% CI 0.2–0.65), the absolute benefit for the country is estimated at 4 fewer cases of pre-eclampsia per 1,000 pregnant women supplemented with calcium (from 5 fewer to 2 fewer) (Table 2). Following the critical appraisal of the review [2] using the AMSTAR tool, we found substantial heterogeneity (I squared >70%: qualitative and quantitative heterogeneity showing inconsistent results) between the trials included in the review. For instance, the multi-country WHO trial included in the review [2] did not demonstrate an effect of calcium in reducing pre-eclampsia (8312 women, RR: 0.92; 95% CI: 0.75–1.13) [12]. A small trial in the review conducted in the Andean/Ecuadorian population with reportedly high frequency of pregnancy-induced hypertension showed large effects of calcium supplementation in reducing pre-eclampsia (106 women, RR: 0.15; 95% CI: 0.04–0.66) [13]. The results from these trials explain the inconsistency in the effect estimates of calcium supplementation in reducing pre-eclampsia. Indeed, small study effects were observed in the effect estimate from the Cochrane Review [2], which might have overestimated the effect of calcium supplementation in pre-eclampsia reduction. This is further complicated by a range of calcium doses (1.5–2 g) that has been evaluated in trials, with supplementation also given at different gestation ages, ranging from 13 to 32 weeks. This may also influence the treatment effect.

**Table 2.** GRADE summary of findings tables based on Cochrane systematic review [2].

Certainty assessment							No. of patients		Effect		Quality of the evidence [22]	Importance
No. of studies	Study design	Risk of bias	Inconsistency	Indirectness	Imprecision	Other considerations	High-dose calcium supplementation (>1 g/day)	No calcium supplementation	Relative (95% CI)	Absolute(95% CI)		
High-dose calcium supplementation (>1 g/day) compared to no calcium supplementation for preventing pre-eclampsia in pregnancy												
**Pre-eclampsia (all women)**												
13	Randomised trials	Not serious	Serious^a^	Not serious	Not serious	Publication bias strongly suspected^b^	379/7851 (4.8%)	510/7879 (6.5%)	RR 0.45 (0.31–0.65)	36 fewer per 1,000 (from 45 fewer to 23 fewer)	⊕⊕○○ Low	Critical
**Pre-eclampsia (women at high risk of pre-eclampsia)**												
5	Randomised trials	Serious^c^	Not serious	Not serious	Not serious	None	9/281 (3.2%)	54/306 (17.6%)	RR 0.22 (0.12–0.42)	138 fewer per 1,000 (from 155 fewer to 102 fewer)	⊕⊕⊕○ Moderate	Critical
**Pre-eclampsia (low-calcium diet)**												
8	Randomised trials	Not serious	Serious^d^	Not serious	Not serious	Publication bias strongly suspected^e^	209/5331 (3.9%)	306/5347 (5.7%)	RR 0.36 (0.20–0.65)	37 fewer per 1,000 (from 46 fewer to 20 fewer)	⊕⊕○○ Low	Critical
**HELLP syndrome**												
2	Randomised trials	Not serious	Not serious	Not serious	Not serious	None	16/6446 (0.2%)	6/6455 (0.1%)	RR 2.67 (1.05–6.82)	2 more per 1,000 (from 0 fewer to 5 more)	⊕⊕⊕⊕ High	Critical
Low-dose calcium supplementation (<1 g/day) compared to no calcium supplementation for preventing pre-eclampsia in pregnancy												
**Pre-eclampsia**												
4	Randomised trials	Very serious^f^	Not serious	Not serious	Not serious	None	24/552 (4.3%)	57/428 (13.3%)	RR 0.36 (0.23–0.57)	85 fewer per 1,000 (from 103 fewer to 57 fewer)	⊕⊕○○ Low	Critical
High-dose vs. low-dose calcium supplementation (<1 g/day) compared to no calcium supplementation for preventing pre-eclampsia in pregnancy												
**Pre-eclampsia**												
1	Randomised trial	Serious^g^	Not serious	oNot serious	Serious^h^	None	7/123 (5.7%)	19/139 (13.7%)	RR 0.42 (0.18–0.96)	79 fewer per 1,000 (from 5 fewer to 112 fewer)	⊕⊕○○ Low	Critical

CI, confidence interval; RR, risk ratio; HELLP. haemolysis, elevated liver enzymes and low platelets.

^a^Serious heterogeneity (I squared = 70%).

^b^Small study effects.

^c^Unclear risk of bias for all the seven domains of bias considered and was published as a letter to the editor (Lopez-Jaramillo et al., 1990).

^d^Serious heterogeneity (I squared = 76%).

^e^Small study effects.

^f^Serious design limitations.

^g^Downgraded by one due to study design limitation.

^h^Downgraded by one due to small study size.

The benefits of calcium supplementation may thus depend upon the trial characteristics such as study size, setting, population, or intervention (timing of supplementation, dose, and frequency). Given the wide variation in effect sizes between trials and overestimation of effects seen in small studies, we downgraded the evidence for desirable effects of calcium in reducing pre-eclampsia to low certainty (Table 1). When applying the evidence to the country context in Nepal, we found the probable desirable effects to be small.

#### Undesirable effects (how substantial are the undesirable anticipated effects?)

The principal undesirable effect of calcium supplementation identified is HELLP (haemolysis, elevated liver enzymes and low platelets) syndrome. HELLP is a serious condition, which when undiagnosed and untreated may pose substantial health risks for both mother and foetus. It is estimated that HELLP syndrome occurs in 0.5–0.9% of pregnancies, and it increases to 10–20% in pregnancy cases with severe pre-eclampsia [14]. The evidence showed that high-dose calcium supplementation increases the risk of HELLP syndrome (*n* = 12,901 women, calcium: 16/6,446, control: 6/6,455, RR: 2.67, 95% CI: 1.05–6.82, high certainty evidence). Applying the evidence to the prevalence of pre-eclampsia in Nepal, high-dose calcium supplementation would be expected to be associated with an estimated 12 more cases of HELLP per 1,000 pregnant women supplemented with calcium [95% CI: 0–41 additional HELLP cases per 1,000 women supplemented with calcium] compared with 4 fewer cases of pre-eclampsia (Table 2). Considering the potential impact, stakeholders raised serious concerns over the occurrence of HELLP syndrome and indicated the need for country-specific evidence for HELLP syndrome associated with calcium supplementation. The syndrome was reported in the studies conducted in populations similar to Nepal such as in India and other countries of Asia. In addition, the pilot study of calcium supplementation in Dailekh district reported that approximately 10% of pregnant women did not complete the full course of supplementation. The reasons for not taking the pill were quoted as illness after taking the calcium pill, difficulty in taking the large-sized tablets, everyday pill burden, fear of side effects, and vomiting [15].

Stakeholders also raised the issue of a potential negative impact of calcium supplementation on the number of women completing the existing iron supplementation programme in Nepal and difficulties in integrating the two schemes. Therefore, we concluded that the undesirable effects were categorised as LARGE, principally due to the increased incidence of HELLP.

#### Certainty of evidence (what is the overall certainty of the evidence of effects?)

The trials including a relatively small number of participants (<1,000) showed a greater effect on pre-eclampsia reduction, whereas trials incorporating a large sample size of over 1,000 participants showed no effect. Therefore, we concluded that there is a low certainty of evidence regarding the desirable effects.

In contrast, the supplementation trials that assessed undesirable effects, including HELLP syndrome, were robust with a relatively large sample size. Taking into account the low certainty evidence of benefit of calcium supplementation but high certainty evidence of undesirable effects, we concluded the overall certainty of the evidence to be moderate. This is primarily because of small study effects (publication bias), inconsistent trials observed for desirable effects, and increased risk of HELLP syndrome reported in high-quality trials.

#### Values (is there important uncertainty about or variability in how much people value the main outcomes?)

A calcium supplementation programme would aim to reduce pre-eclampsia and maternal mortality as the main beneficial outcomes, with adverse events such as HELLP syndrome as the principal harm. Since both pre-eclampsia and HELLP syndrome can result in maternal mortality, these two outcomes would be expected to have similar value judgements among diverse stakeholders, and the ratio between the two outcomes becomes the important factor. For example, a substantial reduction in maternal mortality due to calcium supplementation may result in a minimal increased risk of HELLP syndrome being considered acceptable. However, if the increased risk of HELLP syndrome outweighs the population-level benefit gained from supplementation, the risk would be considered unacceptable. Stakeholders expressed concerns regarding risk communication associated with the adverse effect of calcium supplementation. Their key question was how the message regarding the dosage should be conveyed to pregnant women as based on the evidence that higher doses are associated with the undesirable effect (HELLP syndrome). We therefore judged that there was no important uncertainty for this outcome.

#### Balance of effects (*does the balance between desirable and undesirable effects favour the intervention or the comparison?)*

Given the high certainty of the evidence of increased incidence of HELLP syndrome (undesirable effect) and the small effect size of the desirable effects, coupled with very low certainty of the evidence, the weight of evidence favours the control (no supplementation), rather than the intervention (blanket calcium supplementation). Therefore, we judged that the balance of effects probably favours the comparison (no supplementation).

#### Resources required (how large are the resource requirements (costs)?)

We calculated the approximate cost estimates required for calcium supplementation in the country based on extrapolation of costs from a pilot calcium supplementation programme conducted in Nepal [6]. We calculated costs for high dose (2 g), moderate dose (1.5 g), and dose below the WHO-recommended calcium supplement (1 g) blanket supplementation of calcium (Table 3). A programme providing 1 g of calcium (considered lower dose) would require 2,500 mg of calcium carbonate tablets. Due to the bulk of the tablets, even a lower-dose supplementation programme requires transport and storage at health facilities for significant volumes of bulky tablets, which adds to the costs. We estimated the cost of high-dose calcium supplementation to be approximately 8 million USD and the dose below the WHO-recommended calcium supplement to be 4 million USD, if the programme intends to provide blanket calcium supplementation to all pregnant women in the country in a year. We therefore judged that the resources required are large.

**Table 3.** Potential public health impact in Nepal.

Outcome	Explanation	Illustrative comparative risks^a^ (95% CI)		Relative risk (95% CI)	Absolute (95% CI)
		Assumed risk	Corresponding risk		
		Control	Calcium		
Pre- eclampsia	Applying pooled effect estimate from 2018 systematic review to 2009 health facility Emergency-Obstetrics Care-based prevalence in Nepal	**7 per 1,000**^b^	3 per 1,000 (2–5)	0.45 (0.31–0.65)	**4 fewer per 1,000** (from 5 fewer to 2 fewer)
	Applying high-risk based pooled effect estimate from 2018 systematic review to 2009 health facility Emergency-Obstetrics Care-based prevalence in Nepal (high-dose calcium)	**7 per 1,000**	**3 per 1,000** (2–4)	0.22 (0.12–0.42)	**5 fewer per 1,000** from 6 fewer to 4 fewer)
	Applying low-calcium diet-based pooled effect estimate from 2018 systematic review to 2009 health facility Emergency-Obstetrics Care-based prevalence in Nepal (high-dose calcium)	**7 per 1,000**	**3 per 1,000** **(1–5)**	0.36 (0.20–0.65)	**4 fewer per 1,000** (from 6 fewer to 2 fewer)
	Applying low-dose calcium-based pooled effect estimate from 2018 systematic review to 2009 health facility Emergency-Obstetrics Care-based prevalence in Nepal (below WHO-recommended dose of calcium)	**7 per 1,000**	**3 per 1,000** (2–4)	0.36 (0.23– 0.57)	**4 fewer per 1,000** (from 5 fewer to 3 fewer)
	Applying high-dose vs low-dose calcium-based pooled effect estimate from 2018 systematic review to 2009 health facility Emergency-Obstetrics Care-based prevalence in Nepal	**7 per 1,000**	**3 per 1,000** (1–7)	0.42 (0.18–0.96)	**4 fewer per 1,000** (from 6 fewer to 0 fewer)
HELLP syndrome	Applying pooled effect estimate from 2018 systematic review to 2009 health facility Emergency-Obstetrics Care-based prevalence in Nepal	**7 per 1,000**	**19 per 1,000** (7–48)	2.67 (1.05–6.82)	**12 more per 1,000** (from 0 fewer to 41 more)

^a^We applied effect estimates from the systematic review to a health facility with Emergency-Obstetrics Care-based prevalence of pre-eclampsia in Nepal.

The corresponding risk (and its 95% CI) is based on the assumed risk in the comparison group and the relative effect of the intervention (and its 95% CI).

^b^Based on 2009 prevalence of pre-eclampsia in Nepal.

#### The certainty of the evidence of the required resources (what is the certainty of the evidence of resource requirements (costs)?)

The certainty of the evidence is low regarding resource requirements. A full health economic evaluation is not available. The data we applied to the cost estimates are derived from a pilot supplementation programme implemented in a district with technical support from the USAID-funded Maternal and Child Health Integrated Program [6], which benefitted from synergistic support with other aspects of the programme during implementation and therefore the full implementation costs (direct and indirect) are unlikely to be included. The blanket supplementation of all pregnant women may have higher costs as estimated in Table 3 than targeting only those pregnant women with low calcium intake or at high risk of developing pre-eclampsia. Also, the costs of factors such as management of increased HELLP syndrome to the health system would be large but have not been incorporated in the estimation. Alternative strategies such as strengthening of ANC services with pre-eclampsia prevention interventions may have a lower cost than calcium supplementation. In this regard, the resource estimate is likely to be an underestimate, and we categorised the certainty of evidence regarding the required resources as very low.

#### Cost-effectiveness (does the cost-effectiveness of the intervention favour the intervention or the comparison?)

The Cochrane systematic review did not include cost-effectiveness evaluations of calcium supplementation. A retrospective analysis of the pilot calcium study in Nepal reported only a marginal increase in cost-effectiveness when compared against a scenario without any intervention for managing pre-eclampsia and eclampsia [17]. As per the analysis of this study, the addition of calcium to existing standard of care in Nepal has an 84% probability of cost-effectiveness above a willingness-to-pay threshold of 40 USD when compared to the standard of care alone. However, this threshold is high in the Nepali context where the total per capita spending on health is 58 USD [18].

Primary prevention of pre-eclampsia, such as increasing ANC coverage, routine screening of blood pressure, identifying high-risk women, and examining proteinuria in existing health facilities, may be more cost-effective if these preventive services could be strengthened. Based on the minimal available evidence on cost-effectiveness, we considered the evidence to favour the comparison (no supplementation).

#### Equity (what would be the impact on health equity?)

Blanket supplementation of calcium is unlikely to increase health equity because those who face barriers to access the health facility may also not be able to receive calcium. Women without access to health facilities are generally those in the lowest socioeconomic groups and most likely to have malnutrition, including low-calcium diets during pregnancy.

A blanket supplementation programme may also create health inequity by supplementing women with an adequate dietary calcium intake as well as those with low-calcium diets.

Therefore, we judged that a blanket calcium supplementation programme probably reduces health equity.

#### Acceptability (is the intervention acceptable to key stakeholders?)

Evidence from a small pilot study in Nepal indicated that calcium supplements (1 g/day) are acceptable to pregnant women attending ANC clinics with a compliance rate of 67.3% completing the full course (taking all 300 tablets in 150 days as prescribed) [6]. However, the study reported concerns expressed by the women such as the need to take multiple calcium tablets a day and managing the time intervals for taking calcium and iron–folic acid tablets. Mothers were instructed to take calcium and iron–folic acid separately at different times of the day to avoid the possible negative interaction of calcium with iron supplements. Half of the pregnant women had challenges due to the large size of the calcium tablets and reported difficulty in taking such tablets daily. Given this difficulty, the pregnant women were advised by the Female Community Health Volunteers to split the tablet into two pieces to ease swallowing. The large-sized tablet, duration of intake, and palatability may impact the acceptability of calcium in women.

Stakeholders also raised concerns regarding risk communication to pregnant women. When the high-certainty evidence indicating an increased risk of HELLP syndrome is clearly communicated, it was thought that the pregnant women would be unlikely to accept the supplements.

Stakeholders questioned the high dose (1.5–2 g) of calcium supplemented in the trials, which exceeds the recommended daily dietary allowance of 1,000 mg for pregnant women in Nepal.

There is no national data on dietary calcium intake among pregnant women, but a small study conducted in two districts reported an average intake of 353 mg/day in Nepal [19]. Extrapolating this data nationwide is not possible due to the extremely high demographic and geographic diversity in Nepal. Stakeholders thus reported confusion over the appropriate dose for supplementation. Therefore, it was not possible to draw a definite conclusion regarding acceptability, but with the strongest evidence drawn from the pilot trial, the acceptability of the intervention was categorised as probably yes.

#### Feasibility (is the intervention feasible to implement?)

The supplementation does not seem to be feasible in the current context of Nepal because the intervention requires trained human resources, health infrastructure for transportation and storage capacity, and a regular monitoring mechanism. Universal supplementation of calcium would therefore be resource-intensive compared to the iron/folate supplementation programme, which is implemented nationwide. Calcium supplementation in Nepal would require considering the capacity development needs of health facilities to manage calcium-associated adverse effects and its potential impact on iron absorption. A qualitative study conducted in Western Kenya reported increased workload on the part of the health providers when adding calcium supplementation to an existing iron supplementation programme [20]. Likewise, a pregnant woman in the same study identified food insecurity as one of the barriers to calcium compliance since the instructions suggest taking calcium supplements after taking some food, and a lack of food makes it difficult for a woman to take calcium [20]. Health system challenges in terms of burden to health providers and social parameters such as food insecurity, poverty, and lack of awareness are some of the challenges relevant to Nepal that could impact the operational feasibility of the supplementation programme. Given that Nepal currently implements iron–folic acid supplements to pregnant women, adding calcium would increase the complexity as it would require them to take a minimum of four tablets (2–3 tablets of calcium depending on dose and a tablet of iron–folic acid) per day, thereby maintaining the time duration for taking tablets of calcium and iron [21,22]. It has been recommended by the WHO to take the supplements of iron and calcium separately several hours apart, for example calcium at morning and midday and iron after the evening meal [1]. However, the majority of women with low socioeconomic groups in Nepal have only one or two meals a day.

As a result, such schedules may make it difficult for the women to take supplements as some women reported not being able to find a suitable time for taking supplements and others reported the schedules as inconvenient to follow [15]. All such concerns limit the feasibility of the calcium supplementation programme in Nepal.

Therefore, feasibility is categorised as probably not feasible.

### Recommendation on the use of calcium

Based on the GRADE-EtD evaluation presented in Tables 1 and 4 (GRADE tables), our conclusion was a ‘strong recommendation against the intervention’ of blanket supplementation of calcium in Nepal.

**Table 4.** Cost estimate for blanket supplementation.

	High dose (2 g)	High dose (1.5 g)	Below WHO-recommended dose (1 g) pilot tested in Dailekh	Remarks
Elemental calcium (tablets)	4 tablets of 500 mg each	3 tablets of 500 mg each	2 tablets of 500 mg each	
Calcium carbonate required (weight of supplement)	5,000 mg	3,750 mg	2,500 mg	To fulfil the dose of 500 mg of elemental calcium, 1,250 mg of calcium carbonate is required.
Price per dose (Nepalese rupees, NPR) Curex Pharmaceuticals Nepal (0.016 USD/tablet or NPR 1.35/tablet)	5.4	4.05	2.7	NPR 1.35 per 500 mg. Price of calcium tablets likely to be high when the current inflation rate of (6%) is considered.
Duration of intake (days)	225	225	225	Assuming the timing of supplement initiation at 4 months of pregnancy till the postpartum period
Cost per pregnancy (NPR)	5.4 × 225 = 1215	4.05 × 225 = 911	2.7 × 225 = 608	
Expected pregnancies (N) [16]	758,652	758,652	758,652	If calcium is to be supplemented to all pregnant women (blanket supplementation)
**The total cost of calcium (USD) (approx.)**	**8 million**	**6 million**	**4 million**	**Cost for calcium supplies only**
Logistics costs	High	Moderate	Low	Including storage and transportation cost
Staff training and staff time cost	Staff training for appropriate counselling on calcium supplementation during antenatal care services			

### Justification of the recommendation

We consider that there is a high risk of harm due to increased incidence of HELLP syndrome and potentially reduced compliance with existing iron supplementation, which is not justified by the possibility of a small, if any, absolute reduction in maternal mortality due to pre-eclampsia. In addition, the high resource costs of such a programme would be better applied to strengthening ANC services, including pre-eclampsia screening.

### Subgroup considerations

There is no relevant data about the prevalence of pre-eclampsia in the low-calcium diet subgroup in Nepal or about the prevalence of low-calcium diets. Therefore, it was not possible to conduct a Nepal-specific subgroup analysis.

### Implementation considerations

This analysis does not recommend initiating the implementation of the supplementation.

### Research priorities

Given the weight of evidence against blanket calcium supplementation in Nepal, we would recommend that further research should focus on alternative approaches to reducing maternal deaths due to pre-eclampsia. This should include research on approaches to strengthening antenatal screening services with blood pressure tests and dipstick proteinuria screening tests integrated into routine ANC. Any further trials of calcium supplementation in Nepal should test targeted calcium supplementation in women with low-calcium diets, rather than blanket supplementation. Such trials should include a cost-effectiveness evaluation, given the high resource costs associated with calcium supplementation. Reduction of pre-eclampsia in Nepal is clearly an urgent priority, and alternative interventions to blanket calcium supplementation should be designed and rigorously tested. Moreover, research should prioritise collaboration with the targeted communities to understand their requirements and practices of seeking ANC and their access and utilisation of health services to prevent pre-eclampsia.

## Conclusions

Blanket calcium supplementation in pregnant women is not an appropriate intervention in Nepal based upon this comprehensive context-specific evidence review. Blanket calcium supplementation is unlikely to result in a significant reduction in maternal mortality due to eclampsia, whereas it may cause increased mortality due to HELLP syndrome. Early identification of high-risk pregnant women during their ANC visits in health facilities and case-by-case management of pre-eclampsia would provide an alternative to blanket supplementation. Countries should aim to generate evidence on the calcium intake among women of reproductive age and pregnant women from nationwide surveys. National policy documents regarding calcium supplementation should be revisited based on the available current country-specific evidence.

## Paper context

Pre-eclampsia is a serious cause of maternal death. For prevention, the World Health Organization recommends calcium supplementation for pregnant women. At present, no clear guidelines exist regarding the implementation of this intervention in Nepal. We appraised the global evidence on calcium supplementation for reducing pre-eclampsia and applied GRADE to determine whether blanket supplementation is appropriate in Nepal. Evidence from this study concludes that blanket calcium supplementation is not appropriate for reducing pre-eclampsia in Nepal.
